# Suicidal Ideation Mediates the Relationship Between Affect and Suicide Attempt in Adolescents

**DOI:** 10.3389/fpsyg.2020.524848

**Published:** 2020-12-22

**Authors:** Andrés Rubio, Juan Carlos Oyanedel, Marian Bilbao, Andrés Mendiburo-Seguel, Verónica López, Dario Páez

**Affiliations:** ^1^Facultad de Economía y Negocios, Universidad Andres Bello, Santiago, Chile; ^2^Facultad de Psicología, Universidad Diego Portales, Santiago, Chile; ^3^Facultad de Educación y Ciencias Sociales, Universidad Andres Bello, Santiago, Chile; ^4^Facultad de Psicología, Universidad Alberto Hurtado, Santiago, Chile; ^5^Facultad de Psicología, Pontificia Universidad Católica de Valparaíso, Viña del Mar, Chile; ^6^Faculty of Psychology, University of the Basque Country, San Sebastian, Spain

**Keywords:** positive affect, negative affect, suicidal ideation, suicide attempt, adolescents, positive and negative affect schedule (PANAS)

## Abstract

Suicide, as one of the leading causes of death for the adolescent population, both in Chile and globally, remains a complex and elusive phenomenon. This research studies the association between positive and negative affect in relation with suicidal ideation and suicidal attempt, given that affectivity is a fundamental basis on which people make evaluations on their satisfaction with life. First, it examines the reliability, structure, and validity of Watson’s positive and negative affect scale (PANAS) scale in a representative random sample of Chilean high school students (*N* = 4,568). The scale evidences strong reliability coefficients and a confirmatory factor analysis, excluding one positive (excited) and one negative (nervous) item. The scale shows a satisfactory goodness of fit. Secondly, it investigates the association of PANAS positive and negative affect scores with suicidal ideation as well as reported attempt in adolescents, controlling for the potential effect of age and sex (*N* = 420 high school students). Low positive and high negative affect, but not sex and age, show a significant association with suicidal ideation. Suicidal ideation totally mediates the association of affect with suicide attempt, as expected. Results are discussed regarding prevention and it considers how positive and negative affect can be relevant as indicators for prevention and treatment using widely available technology.

## Introduction

This study examines affect dimensions, measurements, and the association between positive and negative affect regarding suicidal ideation. Almost 800,000 people suicide each year, and in older adolescents it is the second leading cause of death (WHO, 2019). Some studies show that suicidal attempts can be partially explained by the levels of satisfaction with life (Flores et al., 2019) and other hedonic well-being variables such as affects (Sisask et al., 2008; Dunkley et al., 2017). Affectivity is defined based on the “range, intensity, lability, and appropriateness of emotional response” (American Psychiatric Association, 2013, p. 646) experienced by individuals. As has been studied by Watson and Stanton (2017), affect shows a positive versus negative dimension, and on the other hand, it exhibits a high versus low activation or arousal dimension. These structures emerge when analyzing self-reports and other measures of affect. Positive affect implies high (excited, joyful) versus low (bored) arousal positive valence states, while negative affect includes high (annoyed, nervous) versus low (calm, relaxed) arousal valence states (Watson and Stanton, 2017). Positive and negative affect are not opposite but relatively independent dimensions. With the exception of strong negative emotional states, middle-high levels of positive and negative affect may co-exist (Diener et al., 1999; Watson, 2000; Larsen and Prizmic, 2008). The positive and negative affect scale (PANAS) is one of the best-known measures of hedonic well-being and affect balance (Watson et al., 1988; Robles and Páez, 2003; Thompson, 2007). This scale contains 20 mood and emotion descriptors (e.g., active, excited, hostile) which are relatively pure markers of either high negative affect (NA) or high positive affect (PA). Usually the 10 items for positive mood and 10 items for negative mood are aggregated to yield the separate PA and NA scale scores for each subject. Items are usually answered using a five-point scale. In the Spanish language version, in a sample of students from Spain, the Cronbach alpha for NA was 0.85 and 0.88 for PA with the means being 21.67 (*SD* = 6.6) and 34.07 (*SD* = 7.0), respectively (Ramírez-Maestre et al., 2017).

Positive and negative affect are associated with rumination. Studies show an association between rumination and low positive affect (i.e., sadness-depression), while longitudinal studies show more diverse results (Zvolensky et al., 2016; Brookes et al., 2017; Li et al., 2017). Concerning the relation between rumination and negative affect, there is strong evidence for a positive association between rumination and anxiety, but not with other components of high negative affect (Thomsen, 2006). A form of repetitive non-reflective negative rumination or brooding is related to suicidal ideation and negative affect (Surrence et al., 2009; Rogers and Joiner, 2017). Also, suicide-specific rumination has been shown to be related to suicide attempts more than many other common risk factors for suicide (Rogers and Joiner, 2018). Four facets of suicidal ideation and suicide are usually differentiated in the international literature (Nock et al., 2009): passive suicidal ideation (wishing not to live, to be dead, to die), active suicidal ideation (wishing to kill oneself, planning or thinking about how to kill oneself), attempt or autolysis or inflicting harm to try to take one’s life, and consummated suicide or successful attempt to voluntarily take one’s life. It is important to mention that each of these stages corresponds to a different phenomenon, each with a different explanation (Klonsky et al., 2016).

A cross-cultural study in 17 nations found a cross-national lifetime prevalence of 9.2% of passive suicidal ideation, 3.1% of active ideation or plans, and 2.7% of attempts. Across all countries, 60% of transitions from ideation to plan and attempt occur within the first year after ideation onset (Nock et al., 2009). A meta-analysis found a strong but highly heterogeneous association between suicidal ideation and later suicide, *r* = 0.32 (McHugh et al., 2019). Suicidal ideation is associated to affective symptoms in general and amongst young people in particular (Joiner et al., 2005; Van Orden et al., 2010; Teismann et al., 2018; von Brachel et al., 2019). In adolescents hospitalized for psychiatric illness, negative emotions such as anxiety, anger, and hopelessness (Pinto and Whisman, 1996) have a strong relationship with suicidal behaviors. The main issue with this approach is that it is essentially focused on people with psychological or psychiatric disorders (Gould et al., 1998; Prinstein et al., 2000; Sareen et al., 2005; Hauser et al., 2013; Koutek et al., 2016; Barros et al., 2017; Miché et al., 2018; Hill et al., 2019), with less empirical evidence for non-clinical adolescents. Those who have centered research on non-clinical adolescents have found that stress (related to negative affect) and hopelessness and social isolation (associated to low positive affect), are significant predictors of suicidal ideation (Wilburn and Smith, 2005; Van Orden et al., 2010). A meta-analysis based on general population samples found that hopelessness (*r* = 0.31), depression (*r* = 0.24), and anxiety (*r* = 0.16) were three of the five most significant predictors of suicidal ideation (Franklin et al., 2017). This suggests that low positive (related to depression and hopelessness) and high negative affect (associated to both depression and anxiety) are relevant determinants of this type of negative rumination.

In this study, we want to contrast a two-dimensional model of affect and examine its association with satisfaction with life and suicidal ideation. It is expected that positive affect has strong associations with satisfaction with life. Likewise, it is expected that high negative and low positive affect should show a strong association with a form of negative rumination like suicidal ideation, while high positive and low negative affect should show a strong association with functionality and adaptive coping strategies (Yamasaki et al., 2006; Spoor et al., 2007; Nicolas et al., 2014). Finally, we expect that suicidal ideation plays a mediator role between affect and suicide attempt.

## Materials and Methods

This study was approved by the Faculty of Psychology of [BLINDED]. The research was conducted with resources from [BLINDED] projects N° [BLINDED] and [BLINDED]. The principal aim of the study was to determine the relationship between subjective well-being and academic achievement.

### Participants

Research was developed in two steps. First, a probabilistic and stratified sampling was conducted. The sampling units were schools, stratified by type of educational establishment (private, subsidized and public), and by socioeconomic level, from the cities of Santiago, Valparaíso, and Concepción. The sample size was associated with a maximum observed error of ±1.4% assuming a maximum variance and a confidence level of 95%. At the regional level, the absolute error was ±2.4%. In each randomly selected school, all students of second year of high school were surveyed. A total of 4,964 students (50.6% male) answered the questionnaire. Their mean age was 15.59 years (*SD* = 0.823). Students that answered the entire PANAS questionnaire were 4,467 (50.7% male; 11% attrition rate) and their mean age was 15.58 years (*SD* = 0.79).

The second stage aims to study the relationship between suicidal ideation and PANAS, and surveyed students that declared their willingness to participate again in the next academic year (6 months later). From these students (*N* = 503), 420 (35.6% male; 16.60 years (*SD* = 0.59); 16% attrition rate) answered the entire second questionnaire that included suicidal ideation and suicide attempt items.

### Procedures

In both surveys, participation was voluntary. In the first stage, principals and parents were informed. Parents filled a written informed consent form. Each document detailed the study objectives, the institutions responsible for its implementation, confidentiality and how information would be used. It also stated that participation was voluntary and asked for explicit authorization. Each student filled the questionnaire and a professional interviewer in the classroom, from a university research center, was trained in order to explain the questionnaire and answer possible questions. The application was at the end of the academic year and the next application, for the second stage, was at the beginning of the next academic year. In the first application participants answer PANAS and Satisfaction with Life scales.

In the second stage, an online-survey was administered using the Survey Monkey server. Questionnaires were sent to students’ e-mail, including the explanation of the research and an informed consent, which was a request to advance with the questions of the survey. In the second application participation answered PANAS, suicidal ideation scale, and attempted suicide items.

### Measurements

#### Positive and Negative Affect

The Spanish version of PANAS includes 20 adjectives; 10 of them assess Positive Affect (e.g., I feel “excited,” “interested”) and 10 assess Negative Affect (e.g., I feel “scared,” “nervous”). They are rated on five-point Likert scales, ranging from 1 (not at all) to 5 (very much). The participants were asked to report moods and emotions that they had felt during the last week. Separate scores were computed for Positive Affect and Negative Affect. This scale was applied in the first and second questionnaires. PANAS Time 1 with Time 2 disattenuated correlation coefficient score was 0.80 (Time 1: PA *α* = 0.83; NA *α* = 0.81).

#### Satisfaction With Life

The five-item scale developed by Diener et al. (1999) assesses people’s satisfaction with life. It comprises five statements (e.g., “I feel satisfied with my life”) to be rated on a seven-point Likert scale, ranging from 1 (not at all) to 7 (very much). Cronbach’s alpha for this sample was 0.89.

#### Suicidal Ideation Scale

A three-item instrument was developed to assess suicidal thoughts and attempts of young respondents. All the suicidal ideation items were related to active ideation. The three items are: “Have you thought that life was not worth it?,” “Have you ever wished you were dead?,” and “Have you ever thought about ending your life?” during the last academic semester (around 6 months from administration of the instrument); each one was evaluated through a four-point scale, ranging from 1 (never) to 4 (often). Reliability was satisfactory (Cronbach’s *α* = 0.89).

#### Reported Suicide Attempt

It was evaluated by the item “Have you ever attempted to commit suicide?” during the last academic semester (around 6 months from administration of the instrument), with possible answers 1 (never), 2 (once), 3 (a few times), and 4 (many times).

### Analysis

First, descriptive statistics are presented. Then, internal consistency of the scale and subscale are reported. A confirmatory factorial analysis was performed using maximum likelihood as the extraction method. Then, convergent validity was assessed, calculating the correlation between PANAS and satisfaction with life scale. Second, using the online survey, a structural equation model was developed in which positive and negative affect were exogenous variables while suicidal ideation was an endogenous variable. Descriptive analyses, internal consistency, correlations, and exploratory factorial analysis were carried out using the software IBM-SPSS v.20. Confirmatory factorial analysis and structural equation modeling were performed with Mplus v. 6.12. Finally, using Hayes’ procedure (2017), a mediational analysis was carried out, using positive affect and negative affect as predictors; suicidal ideation as mediator; attempt to commit suicide as dependent variable, and age and sex as co-variables. In order to test the mediational effects, we used the SPSS Process macro for bootstrapping indirect effects (Hayes, 2017), which provides indirect effect estimates for mediators, standard errors (SEs), and the confidence intervals (CIs) derived from the bootstrap distribution. Bootstrapped CIs are superior to standard forms of estimating SEs of indirect effects.

## Results

Descriptive statistics are shown in Table 1, where it is possible to distinguish the predominance of positive affect. Positive affect scale has a mean of 3.29 (*SD* = 0.83), while negative affect has a mean of 2.46 (*SD* = 0.82). This difference was statistically significant in the first and second application – in this paper only, second applications analysis are shown [*t* (419) = −9.648, *p* < 0.001]. Gender differences were not significant in positive affect [*t* (418) = −0.887, *p* > 0.05] nor in negative affect [*t* (418) = 1.791, *p* > 0.05]. Age was associated with negative affect (*r* = −0.11, *p* < 0.05).

**Table 1:** Descriptive statistics.

	Sample		Boys		Girls	
	*M*	*SD*	*M*	*SD*	*M*	*SD*
Positive affect	3.29	0.83	3.44	0.78	3.21	0.85
Negative affect	2.46	0.82	2.29	0.82	2.55	0.80
Suicidal ideation	1.78	0.83	1.69	0.71	1.83	0.88

As it can be observed, the scale of suicidal ideation has a mean of 1.78 (*SD* = 0.83). The percentage of participants who answered “never” to each item was 33% for “Have you thought that life was not worth it?,” 42% to “Have you ever wished you were dead?,” and 56% to “Have you ever thought about ending your life?.” Likewise, 80% answered never to “Have you ever attempted to commit suicide.” On the other hand, 8% answered often to “Have you ever thought about ending your life?,” and 4.1% answered many times to “Have you ever attempted to commit suicide?.” Gender differences were not statistically significant *t* (418) = 0.613, *p* > 0.05. Age was not associated with suicidal ideation (*p* > 0.05).

### PANAS Validity

The reliability and internal factor structure of the PANAS was studied through Cronbach’s alpha and confirmatory factor analysis (CFA), to assure its validity. Scale reliabilities were satisfactory, as Cronbach’s alpha was 0.80 for NA and 0.82 for PA (see Tables 2 and 3). If the item “excited” is removed from the positive affect scale, the internal consistency improves (*α* = 0.83) and if the “anxious” item is removed from the negative affect scale, Cronbach’s alpha rises to 0.81.

**Table 2:** Internal consistency analysis of positive affect items.

Positive Affect items	Correlation item total score	Multiple square correlation	Cronbach’s alpha deleting item
PA1Attentive	0.470	0.259	0.814
PA2 Interested	0.455	0.236	0.814
PA3 Alert, awake	0.516	0.284	0.808
PA4 Excited, stimulated	0.329	0.114	0.830
PA5 Enthusiastic	0.604	0.383	0.800
PA6 Inspired	0.544	0.308	0.805
PA7 Proud	0.394	0.168	0.822
PA8 Resolved, decided	0.555	0.322	0.804
PA9 Strong, energetic	0.635	0.492	0.795
PA10 Active	0.648	0.507	0.794

**Table 3:** Internal consistency analysis of negative affect items.

	Correlation item-total score	Multiple square correlation	Cronbach’s alpha deleting item
NA1 Fearful, scared	0.540	0.424	0.785
NA2 Anguished	0.542	0.345	0.784
NA3 Hostile	0.313	0.130	0.809
NA4 Irritable	0.449	0.238	0.795
NA5 Frightened	0.608	0.482	0.778
NA6 Concerned, altered	0.536	0.296	0.785
NA7 Ashamed	0.491	0.283	0.790
NA8 Guilty	0.511	0.277	0.788
NA9 Nervous	0.596	0.384	0.777
NA10 Anxious	0.283	0.136	0.813

**Figure 1:**
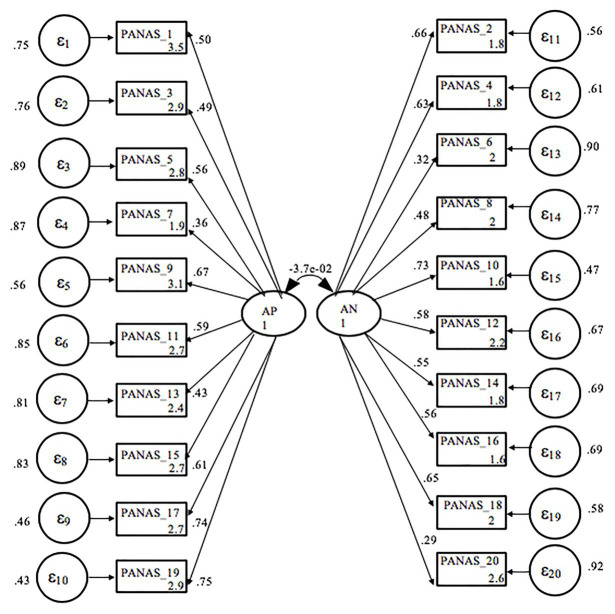
Confirmatory factor analysis including 20 original items and the correlation between positive and negative affect. AP, positive affect; AN, negative affect; *ε_i_* = standard error for each observed variable.

A confirmatory factor analysis (see Figure 1) found that the original two-dimensions model with 20 items shows an unsatisfactory fit with the data: *χ*^2^ = 2363.026, *p* = 0.000; *χ*^2^/degrees of freedom = 13.982; CFI = 0.826; TLI = 0.804, with goodness of fit indices below the conventional 0.90, and RMSEA = 0.076.

**Figure 2:**
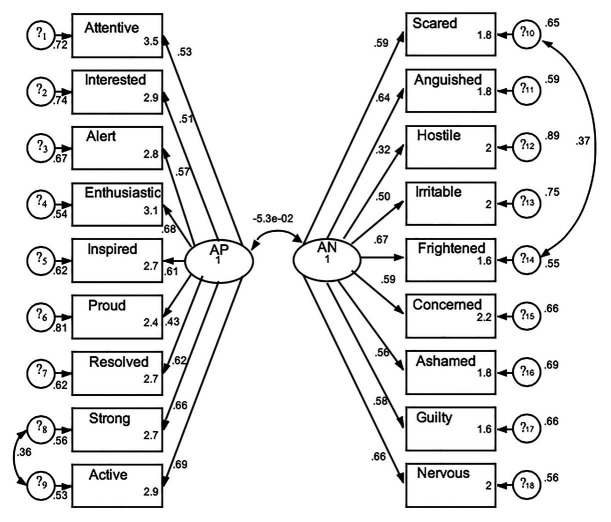
Confirmatory factor analysis including 18 items with better fit and the correlation between positive and negative affect. AP, positive affect; AN, negative affect; *?_i_* = standard error for each observed variable.

In the positive dimension, “excited” and, in the negative dimension, “anxious,” show low loads in the corresponding factors (0.37 and 0.31, respectively), so they were excluded to explore improvements in the data fit, considering also the internal consistency analysis. In addition, considering the modification indexes of the initial model, two pairs of items were covaried, one in the set of positive affects and the other in the set of negative affects. These pairs of items were, respectively, strong-active (affects that usually move together, depending on the mood) and scared-frightened (which in Spanish translate into two verbs that differ little semantically). The results of a second CFA (Figure 2) supported the PANAS two-factor structure; resulting in an 18-item scale with acceptable fit *χ*^2^ = 1062.389, *p* = 0.000; *χ*^2^/degrees of freedom = 8,048; CFI = 0.918; TLI = 0.905; RMSEA = 0.056. The correlation coefficient between the latent variables PA and NA was −0.27 (*p* < 0.001).

Convergent criterion validity was also examined. Affect balance scores were computed as PANAS positive minus PANAS negative. Negative or zero scores were achieved by 19.3% of the sample. This is a reasonable percentage of people with a negative mood state in the last week and shows that around eight participants out of 10 reported a positive affect balance. Affect balance correlates with Diener’s Satisfaction with Life Scale in the first application, with *r* (4450) = 0.50, *p* < 0.001, confirming criterion validity.

### Structural Equation Model and Mediational Model

**Figure 3:**
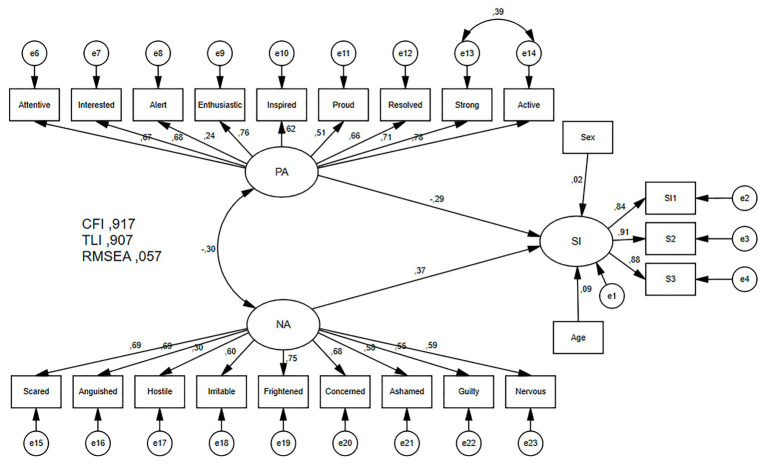
Structural equation model for the relationship between positive affect and negative affect and the association of these variables with suicidal ideation. PA, positive affect; NA, negative affect; SI, suicidal ideation; *e_i_*, standard error for each observed variable. CFI, comparative it index; TLI, Tucker Lewis index; RMSEA, root mean square error of approximation.

The model including suicidal ideation times 2, negative and positive affect times 1, as well as gender and age (see Figure 3), shows a satisfactory goodness of fit: *χ*^2^ = 427.679, *p* = 0.000; *χ*^2^/degrees of freedom = 1.892; CFI = 0.917; TLI = 0.907; RMSEA = 0.057. Positive affect has a negative structural coefficient (−0.29) with suicidal ideation, whereas negative affect shows a stronger positive coefficient (0.37). For both coefficients, low and high confidence interval did not include zero and were significant (low CI −0.41 and high CI −0.15 and low CI 0.26, and high CI 0.48 respectively). Sex and age were not significantly associated with the dependent variable. Finally, the model explains 30% of variance of the endogenous variable.

Finally, a mediational analysis was carried out, using Hayes’ procedure. The analysis considered positive affect and negative affect as predictors, suicidal ideation as mediator, attempt to commit suicide as dependent variable, and age and sex as co-variables. Positive affect correlates *r* = −0.20 with suicidal attempt, *r* = −0.27 with negative affect, *r* = −0.30 with affect balance, and *r* = 0.65 with suicidal ideation, all with *p* < 0.001. An indirect effect is significant if the CI does not include zero values. Ideation (*B* = 0.17) and sex (*B* = −0.14) show significant coefficient predicting suicidal attempt. Ideation mediated the effect of affect on suicide attempt, reducing the coefficient of affect to non-significance (total mediation). Indirect effects of positive and negative affects on suicidal attempt through suicidal ideation are presented (separately) in Table 4:

**Table 4:** Indirect effects of affects on suicidal attempt through suicidal ideation.

	B	SE	BootLLCI	BootULCI
Indirect affect of PA	−0.10	0.03	−0.16	−0.05
Indirect affect of NA	0.17	0.03	0.11	0.24

## Discussion

Regarding the validity of the PANAS scale, it can be said that reliabilities were satisfactory for NA and PA. However, higher reliabilities did not preclude the existence of a different structure from what was expected theoretically. Despite the strong reliabilities of PA and NA, the results of a CFA supported the PANAS two-factor structure only partially. “Excited” in the PA positive dimension and “anxious” in the NA negative dimension showed low loads in the corresponding factors and were excluded to improve data fit. The result of a second CFA using only 18 items supported the PANAS two-factor structure. In relation to criterion validity, PA and NA show a low positive correlation, in agreement with the relative independence of positive and negative affect. As was also expected and confirming criterion validity, affect balance correlates strongly, but below 0.70, with satisfaction with life. Finally, distribution of affect balance scores confirms that 80% show a positive affect balance, in agreement with previous studies of emotional and mood states in the normal population (Watson, 2000; Larsen and Prizmic, 2008; Fredrickson, 2009).

Regarding the report of suicidal behaviors, convergent with previous studies, 8.1% report strong suicidal ideation and 4.1% report clear attempt – similar to 3.1% of plans and 2.7% of attempts in the cross-cultural study by Nock et al. (2009). Regarding the association between affect and suicidal ideation, results are consistent with previous research, indicating that positive and negative emotions are associated with suicidal ideation, although in opposite directions (Hirsch et al., 2007; Bae et al., 2013; Teismann et al., 2019). Negative affect shows a strong association with suicidal ideation, convergent with the strong association of negative affect with rumination. On the other hand, positive affect exhibits a negative association with ideation, confirming that positive emotions play a functional role. Moreover, confirming the relevance of suicidal ideation, this rumination mediates totally the association of affect with suicidal attempt.

The main contribution of this study is that it addresses a specific population segment in which other factors (like bullying) are usually used to approach the problem. The fact that negative affect correlates more strongly with suicidal ideation than positive affect confirms the strong association of negative rumination with anxiety and other high arousal negative emotions usually activated by stress. Low positive affect also shows an association, although weaker, with suicidal ideation. These findings suggest that factors like negative emotional states affect suicidal ideation more strongly than factors like low social integration (which are associated with positive emotional states), as has been previously suggested (Joiner et al., 2005). However, positive affect also shows an important inhibitory role on suicidal ideation and by this path, suicidal attempts decrease. While sex does not affect suicidal ideation, multivariate mediational analysis confirms that women report more suicide attempts compared to men in our sample, congruent with other studies (Kaltiala et al., 1999; Allison et al., 2001; Undheim, 2013). However, meta-analysis done by McHugh et al. (2019) suggests that suicidal ideation is not sensitive enough to be very helpful as a screening test for suicide in non-psychiatric settings. This result shows the limits of suicidal ideation relevance.

This study has clear limitations. It is only based on self-reports, and the suicide attempt indicator was composed of only one item. The attrition rate was high, so we cannot characterize the people who dropped out of the study and potential self-selection bias is a clear limitation. This could have been addressed with an incentive system for participants in the second stage. To better understand the phenomenon in question, it would have been desirable to consider variables that are associated in a relevant way to the problem, such as mental disorders or the sociodemographic characteristics of the participants. This also constitutes a limitation of the study. Finally, another study limitation is social desirability responding, particularly in the specific issue of suicide (Caputo, 2017), where participants could tend to underreport their suicidal behaviors, due to the low acceptance of suicide in Western culture.

In general terms, it can be concluded that when evaluating the health of adolescents (particularly mental health), it is necessary to consider their affective states, especially negative affective states, which are more strongly associated with suicidal ideation and suicide attempts. Following the line of the results of this study and previous studies, special attention should also be paid to the subpopulation of female adolescents, who present a higher frequency in suicide attempts, although male adolescents should not be neglected, because, according to the old and well-known gender paradox in suicide (Canetto and Sakinofsky, 1998), while women have higher rates of suicide attempts, men have higher mortality associated with this phenomenon. Future lines of research should address in more detail the ways in which negative and positive affective states are associated with suicidal ideation; this considering that, in this study, suicidal ideation was the key mediating variable between affect and suicide attempts. Thus, there would be additional elements to develop effective interventions to prevent suicide in adolescents.

## Data Availability Statement

The datasets generated for this study are available on request to the corresponding author.

## Ethics Statement

The studies involving human participants were reviewed and approved by Ethics committee of the School of Psychology of the Pontificia Universidad Católica de Valparaíso. Written informed consent to participate in this study was provided by the participants’ legal guardian/next of kin.

## Author Contributions

AR: processed the experimental data, performed the analysis, drafted the manuscript, and designed the figures. JO and DP: involved in planning and supervised the work, processed the experimental data, performed the analysis, drafted the manuscript, and designed the figures. AM-S: performed the measurements, aided in interpreting the results, and worked on the manuscript. MB and VL: performed the measurements, sample design, aided ininterpreting the results and worked on the manuscript. All authors contributed to the article and approved the submitted version.

### Conflict of Interest

The authors declare that the research was conducted in the absence of any commercial or financial relationships that could be construed as a potential conflict of interest.

## References

[ref1] Allison S. Roeger L. Martin G. Keeves J. (2001). Gender differences in the relationship between depression and suicidal ideation in young adolescents. Aust. N. Z. J. Psychiatry 35, 498–503. 10.1046/j.1440-1614.2001.00927.x, PMID: 11531732

[ref2] American Psychiatric Association (2013). Diagnostic and statistical manual of mental disorders. 5th Edn. Washington, DC: American Psychiatric Pub.

[ref3] Bae S. Lee Y. Cho I. Kim S. Im J. Cho S. (2013). Risk factors for suicidal ideation of the general population. J. Korean Med. Sci. 28, 602–607. 10.3346/jkms.2013.28.4.602, PMID: 23579548 PMC3617315

[ref4] Barros J. Morales S. Echávarri O. García A. Ortega J. Asahi T.. (2017). Suicide detection in Chile: proposing a predictive model for suicide risk in a clinical sample of patients with mood disorders. Braz. J. Psychiatry 39, 1–11. 10.1590/1516-4446-2015-1877, PMID: 27783715 PMC7112738

[ref8] Brookes M. L. Sharpe L. Dear B. F. (2017). Rumination induces a pattern of attention characterized by increased vigilance followed by avoidance of affective pain words. Eur. J. Pain 21, 1197–1208. 10.1002/ejp.1020, PMID: 28272794

[ref9] Canetto S. S. Sakinofsky I. (1998). The gender paradox in suicide. Suicide Life Threat. Behav. 28, 1–23. PMID: 9560163

[ref10] Caputo A. (2017). Social desirability bias in self-reported well-being measures: evidence from an online survey. Universitas Psychologica 16, 245–255. 10.11144/Javeriana.upsy16-2.sdsw

[ref12] Diener E. Suh E. M. Lucas R. E. Smith H. L. (1999). Subjective well-being: encounter of two traditions. J. Pers. Soc. Psychol. 82, 1007–1022.

[ref14] Dunkley D. M. Lewkowski M. Lee I. A. Preacher K. J. Zuroff D. C. Berg J. L.. (2017). Daily stress, coping, and negative and positive affect in depression: complex trigger and maintenance patterns. Behav. Ther. 48, 349–365. 10.1016/j.beth.2016.06.001, PMID: 28390498

[ref16] Flores R. G. Cruz D. M. Quiroz C. O. Fernández-Nistal M. T. (2019). Satisfacción con la vida y uso de sustancias como predictores de intento suicida en adolescentes. Enseñanza e Investigación en Psicología 1, 1–8.

[ref17] Franklin J. C. Ribeiro J. D. Fox K. R. Bentley K. H. Kleiman E. M. Huang X.. (2017). Risk factors for suicidal thoughts and behaviors: a meta-analysis of 50 years of research. Psychol. Bull. 143, 187–232. 10.1037/bul0000084, PMID: 27841450

[ref18] Fredrickson B. (2009). Positivity. New York: Crown Publishers.

[ref19] Gould M. King R. Greenwald S. Fisher P. Schwab M. Kramer R.. (1998). Psychopathology associated with suicidal ideation and attempts among children and adolescents. J. Am. Acad. Child Adolesc. Psychiatry 37, 915–923. 10.1097/00004583-199809000-00011, PMID: 9735611

[ref20] Hauser M. Galling B. Correll C. (2013). Suicidal ideation and suicide attempts in children and adolescents with bipolar disorder: a systematic review of prevalence and incidence rates, risk factors, and targeted interventions. Bipolar Disord. 15, 507–523. 10.1111/bdi.12094, PMID: 23829436 PMC3737391

[ref21] Hayes A. F. (2017). Introduction to mediation, moderation, and conditional process analysis: A regression-based approach. New York: Guilford Publications.

[ref23] Hill R. M. Penner F. Vanwoerden S. Mellick W. Kazimi I. Sharp C. (2019). Interpersonal trust and suicide ideation among adolescent psychiatric inpatients: an indirect effect via perceived burdensomeness. Suicide Life Threat. Behav. 49, 240–252. 10.1111/sltb.12433, PMID: 29370447 PMC8935391

[ref24] Hirsch J. Duberstein P. Chapman B. Lyness J. (2007). Positive affect and suicide ideation in older adult primary care patients. Psychol. Aging 22, 380–385. 10.1037/0882-7974.22.2.380, PMID: 17563193 PMC4846281

[ref25] Joiner T. E. Brown J. S. Wingate L. R. (2005). The psychology and neurobiology of suicidal behavior. Annu. Rev. Psychol. 56, 287–314. 10.1146/annurev.psych.56.091103.070320, PMID: 15709937

[ref28] Kaltiala R. Rimpelä M. Marttunen M. Rimpelä A. Rantanen P. (1999). Bullying, depression, and suicidal ideation in Finnish adolescents: school survey. Br. Med. J. 319, 348–351. 10.1136/bmj.319.7206.348, PMID: 10435954 PMC28187

[ref29] Klonsky E. D. May A. M. Saffer B. Y. (2016). Suicide, suicide attempts, and suicidal ideation. Annu. Rev. Clin. Psychol. 12, 307–330. 10.1146/annurev-clinpsy-021815-093204, PMID: 26772209

[ref30] Koutek J. Kocourkova J. Dudova I. (2016). Suicidal behavior and self-harm in girls with eating disorders. Neuropsychiatr. Dis. Treat. 12:787. 10.2147/NDT.S103015, PMID: 27114709 PMC4833374

[ref31] Larsen R. Prizmic Z. (2008). “The regulation of emotional well-being: overcoming the hedonic treadmill” in The science of subjective well-being. eds. Eid M. Larsen R. J. (New York: Guilford), 258–289.

[ref34] Li Y. I. Starr L. R. Hershenberg R. (2017). Responses to positive affect in daily life: positive rumination and dampening moderate the association between daily events and depressive symptoms. J. Psychopathol. Behav. Assess. 39, 412–425. 10.1007/s10862-017-9593-y

[ref36] McHugh C. M. A. Ryan C. R. Hickie I. B. Large M. M. (2019). Association between suicidal ideation and suicide: meta-analyses of odds ratios, sensitivity, specificity and positive predictive value. Br. J. Psychol. Open 5:e18. 10.1192/bjo.2019.15, PMID: 30702058 PMC6401538

[ref37] Miché M. Hofer P. D. Voss C. Meyer A. H. Gloster A. T. Beesdo-Baum K.. (2018). Mental disorders and the risk for the subsequent first suicide attempt: results of a community study on adolescents and young adults. Eur. Child Adolesc. Psychiatry 27, 839–848. 10.1007/s00787-017-1060-5, PMID: 29027588 PMC6013520

[ref38] Nicolas M. Martinent G. Campo M. (2014). Evaluation of the psychometric properties of a modified positive and negative affect schedule including a direction scale (PANAS-D) among French athletes. Psychol. Sport Exerc. 15, 227–237. 10.1016/j.psychsport.2014.01.005

[ref39] Nock M. K. Hwang I. Sampson N. Kessler R. C. Angermeyer M. Beautrais A.. (2009). Cross-national analysis of the associations among mental disorders and suicidal behavior: findings from the WHO world mental health surveys. PLoS Med. 6:e1000123. 10.1371/journal.pmed.1000123, PMID: 19668361 PMC2717212

[ref43] Pinto A. Whisman M. (1996). Negative affect and cognitive biases in suicidal and nonsuicidal hospitalized adolescents. J. Am. Acad. Child Adolesc. Psychiatry 35, 158–165. 10.1097/00004583-199602000-00008, PMID: 8720625

[ref44] Prinstein M. Boergers J. Spirito A. Little T. Grapentine W. (2000). Peer functioning, family dysfunction, and psychological symptoms, in a risk factor model for adolescent inpatients’ suicidal ideation severity. J. Clin. Child Psychol. 29, 392–405. 10.1207/S15374424JCCP2903_10, PMID: 10969423

[ref46] Ramírez-Maestre C. Correa M. Rivas T. López-Martínez A. E. Serrano-Ibáñez E. R. Esteve R. (2017). Psychometric characteristics of the Flourishing Scale-Spanish Version (FS-SV). The factorial structure in two samples: students and patients with chronic pain. Personal. Individ. Differ. 117, 30–36. 10.1016/j.paid.2017.05.035

[ref47] Robles R. Páez F. (2003). Estudio sobre la traducción al español y las propiedades psicométricas de las escalas de afecto positivo y negativo (PANAS). Salud Mental 26, 69–75.

[ref48] Rogers M. L. Joiner T. E. (2017). Rumination, suicidal ideation, and suicide attempts: a meta-analytic review. Rev. Gen. Psychol. 21, 132–142. 10.1037/gpr0000101

[ref49] Rogers M. L. Joiner T. E. (2018). Suicide-specific rumination relates to lifetime suicide attempts above and beyond a variety of other suicide risk factors. J. Psychiatr. Res. 98, 78–86. 10.1016/j.jpsychires.2017.12.017, PMID: 29304348

[ref50] Sareen J. Cox B. Afifi T. de Graaf R. Asmundson G. ten Have M.. (2005). Anxiety disorders and risk for suicidal ideation and suicide attempts: a population based longitudinal study of adults. Arch. Gen. Psychiatry 62, 1249–1257. 10.1001/archpsyc.62.11.1249, PMID: 16275812

[ref52] Sisask M. Värnik A. Kõlves K. Konstabel K. Wasserman D. (2008). Subjective psychological well-being (WHO-5) in assessment of the severity of suicide attempt. Nord. J. Psychiatry 62, 431–435. 10.1080/08039480801959273, PMID: 18846444

[ref55] Spoor S. T. Bekker M. H. Van Strien T. van Heck G. L. (2007). Relations between negative affect, coping, and emotional eating. Appetite 48, 368–376. 10.1016/j.appet.2006.10.005, PMID: 17145096

[ref56] Surrence K. Miranda R. Marroquin B. M. Chan S. (2009). Brooding and reflective rumination among suicide attempters: cognitive vulnerability to suicidal ideation. Behav. Res. Ther. 47, 803–808. 10.1016/j.brat.2009.06.001, PMID: 19577225

[ref57] Teismann T. Brailovskaia J. Margraf J. (2019). Positive mental health, positive affect and suicide ideation. Int. J. Clin. Health Psychol. 19, 165–169. 10.1016/j.ijchp.2019.02.003, PMID: 31193136 PMC6517639

[ref58] Teismann T. Forkmann T. Brailovskaia J. Siegmann P. Glaesmer H. Margraf J. (2018). Positive mental health moderates the association between depression and suicide ideation: a longitudinal study. Int. J. Clin. Health Psychol. 18, 1–7. 10.1016/j.ijchp.2017.08.001, PMID: 30487904 PMC6220923

[ref59] Thompson E. R. (2007). Development and validation of an internationally reliable short-form of the positive and negative affect schedule (PANAS). J. Cross-Cult. Psychol. 38, 227–242. 10.1177/0022022106297301

[ref60] Thomsen D. T. (2006). The association between rumination and negative affect: a review. Cognit. Emot. 20, 1216–1235. 10.1080/02699930500473533

[ref61] Undheim A. (2013). Involvement in bullying as predictor of suicidal ideation among 12- to 15-year-old Norwegian adolescents. Eur. Child Adolesc. Psychiatry 22, 357–365. 10.1007/s00787-012-0373-7, PMID: 23361192

[ref62] Van Orden K. Witte T. Cukrowicz K. Braithwaite S. Selby E. Joiner T. (2010). The interpersonal theory of suicide. Psychol. Rev. 117, 575–600. 10.1037/a0018697, PMID: 20438238 PMC3130348

[ref63] von Brachel R. Teismann T. Feider L. Margraf J. (2019). Suicide ideation as a predictor of treatment outcomes in cognitive-behavioral therapy for unipolar mood disorders. Int. J. Clin. Health Psychol. 19, 80–84. 10.1016/j.ijchp.2018.09.002, PMID: 30619501 PMC6300714

[ref65] Watson D. (2000). Mood and temperament. New York: Guilford Press.

[ref66] Watson D. Clark L. A. Tellegen A. (1988). Development and validation of brief measures of positive and negative affect: the PANAS scale. J. Pers. Soc. Psychol. 54, 1063–1070. 10.1037/0022-3514.54.6.1063, PMID: 3397865

[ref67] Watson D. Stanton K. (2017). Emotion blends and mixed emotions in the hierarchical structure of affect. Emot. Rev. 9, 99–104. 10.1177/1754073916639659

[ref69] Wilburn V. Smith D. (2005). Stress, self-esteem, and suicidal ideation in late adolescents. Adolescence 40, 33–45. PMID: 15861616

[ref100] WHO (2019). Meltal health and substance use. Available at: https://www.who.int/teams/mental-health-and-substance-use/suicide-data#:~:text=Close%20to%20800%20000%20people,and%20occurs%20throughout%20the%20lifespan (Accessed June 7, 2019).

[ref71] Yamasaki K. Sakai A. Uchida K. (2006). A longitudinal study of the relationship between positive affect and both problem-and emotionfocused coping strategies. Soc. Behav. Personal. Int. J. 34, 499–510. 10.2224/sbp.2006.34.5.499

[ref72] Zvolensky M. J. Paulus D. J. Bakhshaie J. Garza M. Ochoa-Perez M. Lemaire C.. (2016). Interactive effect of negative affectivity and rumination in terms of mental health among Latinos in primary care. J. Racial Ethn. Health Disparities 3, 646–657. 10.1007/s40615-015-0183-y, PMID: 27294754 PMC11862904

